# Circulating Serum Amyloid A, hs-CRP and Vitamin D Levels in Postmenopausal Osteoporosis 

**DOI:** 10.31661/gmj.v8i0.1548

**Published:** 2019-10-09

**Authors:** Anahid Safari, Afshin Borhani-Haghighi, Mehdi Dianatpour, Seyed Taghi Heydari, Farzaneh Foroughinia, Gholamhossein Ranjbar Omrani

**Affiliations:** ^1^Stem Cells Technology Research Center, Shiraz University of Medical Sciences Shiraz, Iran; ^2^Clinical Neurology Research Center, Shiraz University of Medical Sciences, Shiraz, Iran; ^3^Health Policy Research Center, Institute of Health, Shiraz University of Medical Sciences, Shiraz, Iran; ^4^Clinical Pharmacy Department, Shiraz University of Medical Sciences, Shiraz, Iran; ^5^Endocrinology & Metabolism Research Center, Shiraz University of Medical Sciences, Shiraz, Iran

**Keywords:** Osteoporosis, Postmenopausal, Vitamin D, C-Reactive Protein, Serum Amyloid A Protein, Bone Density

## Abstract

**Background:**

Both vitamin D and inflammation were investigated as important players in the pathogenesis of postmenopausal osteoporosis. This study compared vitamin D, inflammatory the biomarkers serum levels and their association with bone mineral density (BMD) in case and control groups to evaluate the possible immune-regulatory effect of vitamin D in this population.

**Materials and Methods:**

Participants in post-menopausal age, were categorized to 44 osteoporotic vs. 44 healthy aged-matched women according to WHO criteria. Total BMD, T- scores, Z-scores as well as fracture risk were measured in both groups, using Hologic system Dual-energy X-ray absorptiometry (DEXA). Serum 25-OH vitamin D, high sensitive CRP (hs-CRP) and serum amyloid A (SAA) were compared between groups. The association between serum biomarkers level and BMD were also investigated. The same evaluations were performed for vitamin D deficient (<20 ng/mL) and non-deficient (≥20 ng/mL) subgroups.

**Results:**

Vitamin D deficiency was higher in the osteoporotic group (32.6%) in comparison with the control group (25.6%), but the differences were not significant (P=0.47). There were no significant differences in serum levels of hs-CRP and SAA (P=0.83 and P=0.39) as well. No significant association between serum inflammatory biomarkers, vitamin D, and BMD were detected (P≥0.05). The results were the same for vitamin D deficient and non-deficient subgroups (P≥0.05).

**Conclusion:**

In the current study, the beneficial effects of vitamin D as a result of its immune-regulatory mechanisms was not reached. Larger scale studies might pave the way to define vitamin D benefits in postmenopausal osteoporosis.

## Introduction

Osteoporosis and its related complications are global health issues. Women in the menopausal phase are known as one of the important population at risk [1]. Diagnosis and treatment of vitamin D deficiency as a worldwide phenomenon is a global concern [2, 3]. Although there are growing number of evidences, indicating vitamin D deficiency as a risk factor for osteoporosis [4-6], the association between vitamin D serum level and bone mineral density (BMD) as the most reliable diagnostic criteria for osteoporosis remains to be confirmed [7]. Even though some studies have stated the association between low serum 25-OH vitamin D [25(OH)D] level and low BMD [8, 9], others failed to exhibit this association [10-12]. Association between inflammatory biomarkers and osteoporosis is the other ambiguous issue that has to be addressed [13], where some results are supportive [14, 15] while others are against it [16, 17]. Hence, whether or not inflammation can be defined as etiology for osteoporosis or is it in association with other etiologies, remains to be answered [18]. In recent years, vitamin D immune-regulatory mechanism of action in various kind of diseases has attracted vigorous attention [19, 20]; however, there are few reports on osteoporosis [21] as an immune-based disorder [22]. With respect to postmenopausal osteoporosis, data are even scarce [23]. From a wide range of inflammatory biomarkers, high-sensitivity CRP (hs-CRP) is widely used as a credible acute phase reactant, since it can detect the minimal rise in CRP level, even under 10 mg/L, which is not usually detectable by CRP [24]. Recently, serum amyloid A (SAA) was suggested to have advantages in the quantitative assessment of inflammatory responses. Also, new experimental studies have revealed bone regulatory effects for this member of the apoprotein family (SAA3) by modulating osteoblasts and osteoclasts function [25]. As far as we know, the association between this new inflammatory and bone regulatory biomarker and osteoporosis has been investigated, merely in a few human studies (SAA1), which have mainly worked on gene polymorphisms of SAA in a special population [26]. The association between inflammatory biomarkers, vitamin D status, and BMD should also be studied in postmenopausal women to reveal the possible immune-mediated mechanisms of vitamin D in this population. In the current study, we compared 25(OH)D, hs-CRP and SAA levels plus their association with BMD in postmenopausal osteoporotic women in comparison with their healthy matched controls to evaluate the protective effect of vitamin D, particularly through its anti-inflammatory mechanisms.

## Materials and Methods

This prospective case-control study was conducted in Namazi hospital affiliated with Shiraz University of Medical Sciences from June 2017 till May 2018. This study was approved by the institutional review board (IRB) of Shiraz University of Medical Sciences (No#1397-01-67-14348), written informed consent was obtained from each participant. If participants were severely ill, consent was obtained from their family members. Total of 44 patients and 44 controls were recruited according to α=0.05, β=0.80, and the effect size of 0.6 calculated by G*power Software (Release 3.1.9.3, Heinrich-Heine-Universität, Düsseldorf,Germany). Initially, 254 women in menopausal age were assessed, but after considering all the inclusion and exclusion criteria, 44 women with defined criteria of osteoporosis were enrolled. Also, 44 healthy age-matched women in the postmenopausal period were recruited as controls. Participants were also categorized into vitamin D deficient (<20 ng/mL) and non-deficient (≥20 ng/mL) subgroups according to their serum vitamin D level. The inclusion and exclusion criteria, as well as criteria for selecting the control group, were as follows: Osteoporotic women with at least 2-years history of menopause who had signed the written informed consent were included. The diagnostic criteria for osteopenia/osteoporosis was defined according to WHO criteria (T-score: > -1: normal, -1 to -2.49: osteopenia and ≤ -2.5: osteoporosis) [27]. Patients with osteoporosis risk factors, such as prolactinoma, Cushing syndrome, hyperparathyroidism, celiac disease, malabsorption, total parenteral nutrition, immobile patients and women with a history of gastrectomy, ovariectomy or hysterectomy were excluded. Other exclusion criteria were as follows: conditions that affect vitamin D, hs-CRP, and SAA such as febrile illnesses, any proven neoplastic, rheumatologic or inflammatory diseases, acute or chronic kidney diseases, liver or respiratory failure, recent surgery, thyroid and parathyroid disfunction, Alzheimer, and cerebrovascular diseases. Those who were receiving vitamin D or calcium supplements, osteoporosis treatments, calcitonin, parathyroid hormone, glucocorticoids, thiazide diuretics, anticoagulants, LH-RH agonist, anticonvulsants, pioglitazone, methotrexate, and aluminum-containing anti-acid were also excluded. Age-matched women with normal BMD, and at least 2-years history of menopause who met the inclusion criteria were recruited as the control. For Laboratory investigations, total of 10cc peripheral blood was taken from each participant. Then, the sera were separated from cells via centrifugation at 3000 rpm for 10 min. The sera were stored at -80°C and then thawed. Finally, calcium, phosphor, alkaline phosphatase (ALP), fasting blood sugar (FBS), blood urea nitrogen (BUN), creatinine (Cr) and lipid profile (total cholesterol, low-density lipoprotein [LDL], high-density lipoprotein [HDL]) were assessed by routine laboratory methods for both case and control groups. The 25-OH vitamin D (KAP1971, DIAsource Immuno Assays S.A., Louvain-la-Neuve, Belgium), SAA level (KHAoo11, Invitrogen, California, US), and hs-CRP (4360, Diagnostics Biochem Canada, Ontario, Canada) were measured in serum samples of post-menopausal women by ELISA method using calibration solutions. Serum biomarkers concentrations were reported according to the standard curves made by the Optical Densities (at 450 nm) and standard solutions concentration. Hyperlipidemia (HLP) was defined as current or positive history, fasting total cholesterol level>200 mg/dL, LDL>130 mg/dL and/or fasting triglycerides level>180 mg/dL [28]. Hypertension (HTN) was defined as a positive history, systolic blood pressure of 140mmHg and/or diastolic pressure>90 mmHg, electrocardiographic or retinal sign of hypertension [29]. Diabetes mellitus (DM) was defined as having a positive history and/or Hb A1C more than seven [30]. Inclusion and exclusion criteria and history of DM, HLP, and HTN were defined, using a reliable questionnaire. BMD measurements were performed for both postmenopausal groups, using Hologic system DEXA (Dual-energy X-ray absorptiometry, Discovery QDR, USA). Total BMD, T- scores, and Z-scores in the lumbar spine and hip, as well as fracture risk (hip and major osteoporotic), were measured in all the participants. Statistical analyses were carried out using SPSS (IBM, USA), version 20. In normally distributed groups, the results are presented as mean and 95% confidence interval. Chi-square and independent t-test were applied to test the differences in variables. Pearson correlation coefficient was used to determine the association between hs-CRP, SAA, and Vitamin D levels as well as osteoporosis diagnosis scales (BMD, T-score, Z-score, and fracture risk) in both groups. The significance level in this study was considered at 0.05.

## Results

From the 254 women with at least 2-years history of menopause, 44 osteoporotic women vs. 44 healthy age-matched women were recruited after considering all the inclusion and exclusion criteria. Subgroups were selected according to vitamin D serum level: deficient (<20 ng/mL) and non-deficient (≥20 ng/mL). Table-1 shows the participants demographic variables, osteoporosis diagnosis criteria, and risk factors. Vitamin D, hs-CRP, SAA levels, and other laboratory variables are also shown in Table-1. Body mass index (BMI) and history of exercise were significantly different between the case and control groups, but the history of smoking, as well as DM, HTN or HLP, were not significantly different (P≥0.05, Table-1). There was no significant difference in SAA, hs-CRP, and vitamin D level as well as calcium level and other laboratory variables between case and control groups (P≥0.05, Table-1). Vitamin D deficiency was higher in the case group (32.6%) in comparison with the control group (25.6%), but the differences were not significant (P=0.47). There were no significant differences in hs-CRP and SAA levels between vitamin D deficient (<20 ng/mL) and non-deficient (≥20 ng/mL) participants in both case and control groups (P≥ 0.05). By evaluating all participants, hs-CRP and SAA levels were not significantly different (Table-2). Amongst postmenopausal women with vitamin D level <20 ng/mL, hs-CRP and SAA levels were higher in the osteoporotic compared to non-osteoporotic group; however, the differences were not significant (P=0.42 and P=0.76, Table-3). Amongst postmenopausal women with vitamin D level ≥20 ng/m, there was no significant difference in serum levels of hs-CRP and SAA between the case and control groups (P=0.83 and P=0.43, Table-3). There were no significant associations between hs-CRP (R= - 0.28, R= - 0.02 and P=0.0, P=0 .88, respectively), SAA ( R= - 0.06, R= - 0.14 and P=0.72, P=0.38, respectively), and vitamin D level in both case and control groups as well as these serum biomarkers and BMD measurements (Total BMD, T-score, Z- score and fracture risk, P>0.05, Table-4). Only a significant association was observed between hs-CRP and SAA levels in case and control groups (R=36, R=32 and P=0.02, 0.03, respectively). These results were the same for vitamin D subgroups (results are not shown).

## Discussion

In the current study, BMI and history of exercise were significantly different between groups. Although vitamin D deficiency was higher in the osteoporotic group, the differences were not significant. The results also showed no significant differences in hs-CRP and SAA levels between groups. In addition, there were no significant associations between serum levels of inflammatory biomarkers and vitamin D as well as total BMD, T-score, Z- score, and fracture risk in both groups. The results were the same, when participants were categorized according to their vitamin D level.

Previous studies have investigated the beneficial effect of vitamin D in osteoporosis, regardless of its mechanisms. One of these reports contradict the preventive role of vitamin D alone in osteoporosis as well as its related risk factors such as postmenopausal phase or those receiving glucocorticoids [31]. Their reports can support our results.In addition, in line with our results, some other studies reported no association between serum vitamin D level and BMD in osteoporosis [11, 12]. There are also some reports against this association in postmenopausal osteoporosis that can support our results better. Labronici *et al*. investigated vitamin D level and bone mineral density in 250 postmenopausal women and obtained no significant difference in vitamin D level in the subgroups as well as no significant correlation between vitamin D levels and BMD [32]. Hosseinpanah *et al*. study also confirms our findings in an Iranian population by revealing no significant association between 25(OH)D and BMD in 245 healthy free-living postmenopausal women [10].Regarding the association between inflammatory biomarkers and bone mass, some studies revealed no independent association between hs-CRP level and bone mass [33-35], which are in line with our results. However, data on SAA and postmenopausal osteoporosis are insufficient. As far as we know, there is only one study and it did not consider the association between SAA level and BMD [26].In contrast to our results, there are some reports in favor of the association between BMD and serum vitamin D [8, 9, 36] or hs-CRP [37] in osteoporosis, but the data are inadequate with respect to postmenopausal osteoporosis.To sum up, it is difficult to confirm the protective role of vitamin D (alone) and its immune-regulatory mechanism of action in osteoporosis and its related conditions, due to diversity in studies and contradictive results. These differences might have been influenced by several factors. Christodoulou *et al*. [7] suggested study design and population (age, gender, and osteoporosis predisposing conditions) as important factors. Diversity in Vitamin D dosage and administrative methods should also be considered in the interventional studies.Considering the present evidences, designing large scale investigations with specific focus on the unanswered questions might be feasible. We eliminated all the confounding factors that might have affected vitamin D, hs-CRP and SAA levels as well as BMD such as infection, recent surgery, neoplastic, and inflammatory diseases, renal or hepatic failure, Parkinson, Alzheimer, and cerebrovascular diseases in addition to any drugs or supplements (vitamin D, calcium) affecting serum levels of biomarkers or BMD. This should be considered as a remarkable advantage of the current study. One of our major limitations was to find a postmenopausal population with the exclusion of the above-mentioned confounding factors. At the same time, finding non osteoporotic women with the defined criteria was even harder. The wide range of exclusion criteria might increase the reliability of the results, but at the same time it could lead to small sample size, which in turn can be assumed as a possible cause for negative results. Even though might be so, it seems logical to relay on the results due to the following advantages, such as focusing on a pure study population, considering a control group with the exact similar criteria to compare the results as well as standard study design. In addition, we could not find any similar study that could reject our findings.

## Conclusion

In conclusion, our results did not confirm the significant role of inflammatory biomarkers in postmenopausal osteoporosis. Beneficial effect of vitamin D due to its immune-regulatory mechanisms could not be proven. Larger scale studies might pave the way to reveal vitamin D protective role in post-menopausal osteoporosis.

## Acknowledgement

We would like to thank Mr. Neydavoodi at clinical neurology research center of Shiraz University of Medical Sciences for statistical analysis.

## Conflict of Interest

The authors have no conflict of interest.

**Table 1:** Comparison between Demographic, Laboratories, Osteoporosis Diagnosis Criteria, and Risk Factors in Osteoporotic and Healthy Age-Matched Postmenopausal Women

**Variables**	**Case group** **(n=44, mean, CI)**	**Control group** **(n=44, mean, CI)**	**P-value**
**Age**	64.22 (59.88-68.30)	63.67(61.80-65.69)	0.33
**BMI**	30.47 (28.82-32.33)	27.52 (26.03-28.92)	0.015
**Exercise**	1 (2.32%)	11 (26.23%)	0.002
**Smoking**	2 (4.73%)	1 (2.46%)	0.57
**HLP**	4 (9.37%)	6 (14.34%)	0.47
**DM**	2 (4.77%)	3 (7.08%)	0.64
**HTN**	2 (4.76%)	4 (9.38%)	0.39
**Systolic blood pressure (mmHg)**	121.89 (117.81-126.15)	125.12 (119.73-131.19)	0.36
**Diastolic blood pressure (mmHg)**	78.65 (74.69-80.25)	77.32 (74.69-80.25)	0.48
**hs-CRP(ng/ml)**	3251.33(2583.483957.42)	3157.97 (2579.69-3772.91)	0.83
**SAA(ng/ml)**	419.25 (319.30-525.55)	360.07 (280.13-442.16)	0.39
**25-OHvitamin D (ng/ml)**	32.26 (25.35-39.64)	31.62 (26.95-36.60)	0.88
**Calcium (mg/dl)**	9.14 (9-9.27)	9.11 (8.97-6.26)	0.79
**Alkaline phosphatase (mg/dl)**	161.77 (147.46-175.77)	144.91 (134.53-155.13)	0.06
**FBS (mg/dl)**	106.30 (101.90-111.45)	105.79 (102.21-109.19)	0.86
**Triglyceride (mg/dL)**	102.09 (91.30-113.60)	112.05 (102.16-123.41)	0.20
**Cholesterol (mg/dL)**	213.40 (202.73-224.47)	221.21 (208.82-234.41)	0.38
**HDL (mg/dL)**	59.16 (56.03-62.67)	58.56 (55.35-61.85)	0.80
**LDL (mg/dL)**	107.67 (100.58-115.15)	114.51 (106.15-123.46)	0.24
**Total BMD (spine)**	0.674 (0.65-.70)	0.98 (0.96-1.01)	<0.001
**Total T-score (spine)**	-3.39 (-3.64- -3.13)	-0.57 (-0.76 - -0.36)	<0.001
**Total Z-score (spine)**	-1.73 (-1.96 - -1.47)	0.64 (0.45-0.86)	<0.001
**Total BMD (hip)**	0.67 (0.64-0.70)	0.98 (0.96-1.01)	<0.001
**Total T-score (hip)**	-2.21 (-2.46 - -1.95)	0.33 (0.10-0.56)	<0.001
**Total Z-score (hip)**	-1.11 (-1.32 - -.86)	1.13 (0.92-1.34)	<0.001
**Fracture risk (hip)**	3 (2.24- 3.87)	0.18 (0.14-0.23)	<0.001
**Fracture risk**	7.78) 5.83-10.25(	1.82 )1.63-2.06(	<0.001

**CI:** Confidence interval; **BMD:** Bone mineral density; **BMI:** Body mass index; **HLP:** Hyperlipidemia; **DM:** Diabetes mellitus; **HTN:** Hypertension; **FBS:** Fasting blood sugar;** LDL:** Low-density lipoprotein; **HDL:** High-density lipoprotein; **SAA:** Serum amyloid A

**Table 2:** hs-CRP and SAA Levels Were Compared in All Participants According to Serum Vitamin D Level

**Serum biomarker** **(ng/mL)**	**Vitamin D level (ng/mL)**	**Mean± SD**	**Confidence interval**	**P-value**
hs-CRP	<20	3181.40± 1896.54	(2491.19-3969.44)	0.94
	≥20	3214.18± 2196.40)	(2706.75-3797.71)	
SAA	<20	417.36± 375.03	(274.54-571.85)	0.61
	≥20	378.32 ± 301.48	(303-460.96)	

**Table 3:** hs-CRP and SAA Levels Were Compared in Case and Control Groups According to Serum Vitamin D Level

**Vitamin D level**	**Serum biomarker (ng/mL) **	**Groups**	**Mean (CI)**	**P-value**
<20 (ng/mL)	hs-CRP	Control	2827.88 (2113.25-3596.48)	0.42
		Case	3459.16 (2366.72-4817.18)	
	SAA	Control	391.08 (198- 602.15)	0.76
		Case	438.07 (244.18-637.10)	
≥20 (ng/mL)	hs-CRP	Control	3271.43 (2482.82-4033.91)	0.83
		Case	3151 (2372.06-3952.53)	
	SAA	Control	349.41 (263.09-441.04)	0.43
		Case	410.20 (293.82-539.46)	

**Table 4:** Association between Inflammatory Biomarkers, Vitamin D, and BMD Measurements in Case and Control Groups

**Serum biomarkers**			**Spine**			**Femur**			**Fracture risk**		
			**Total** **BMD**	**Total** **T-score**	**Total** **Z-score**	**Total** **BMD**	**Total T-score**	**Total Z-score**	**Hip**	**Major osteoporotic**
hs-CRP	Case	R	0.24	0.24	0.10	0.13	0.14	0.006	-0.02	-0.05
		P	0.13	0.12	0.53	0.41	0.38	0.97	0.89	0.74
	Control	R	0.06	0.06	0.14	0.23	0.24	0.31	-0.06	-0.22
		P	0.72	0.70	0.39	0.13	0.12	0.052	0.68	0.16
SAA	Case	R	0.06	0.06	-0.04	-0.04	0.03	-0.13	-0.11	-0.05
		P	0.69	0.71	0.82	0.80	0.83	0.39	0.48	0.75
	Control	R	0.09	0.08	0.20	0.14	0.14	0.24	0.11	0.02
		P	0.57	0.61	0.18	0.36	0.39	0.11	0.47	0.89
Vitamin D	Case	R	0.05	0.05	0.18	-0.14	-0.15	-0.06	0.14	0.30
		P	0.74	0.77	0.26	0.37	0.34	0.72	0.36	0.07
	Control	R	-0.07	-0.06	-0.14	0.18	0.19	0.14	-0.22	-0.13
		P	0.66	0.67	0.39	0.24	0.22	0.38	0.16	0.41
